# Young Man With Non-hypertensive Ascites of Unexpected Cause: When Ockham’s Razor Is Not Sufficient

**DOI:** 10.7759/cureus.25385

**Published:** 2022-05-27

**Authors:** Julián Rondón-Carvajal, Jose C Alvarez-Payares, Natalia Arias-Madrid, Jeanneth Echeverri-Villegas, Laura Uribe-Zapata

**Affiliations:** 1 Internal Medicine, University of Antioquia, Medellín, COL; 2 Internal Medicine, Leon XIII Clinic, IPS (Institución Prestadora de Salud) Universitaria, Medellín, COL; 3 General Surgery, University of Antioquia, Medellín, COL; 4 Pathology, University of Antioquia, Medellín, COL; 5 Medicine, Cooperative University of Colombia, Medellín, COL

**Keywords:** cancer, tuberculosis, peritoneal carcinomatosis, peritoneum, ascites

## Abstract

Ascites is defined as the accumulation of fluid in the peritoneal cavity, following an imbalance between production and reabsorption; it is detectable from 50 mL on ultrasound. Three mechanisms have been classically implicated, according to Starling's forces: an increase in the hydrostatic pressure gradient (increased portal venous pressure), a reduction in the oncotic pressure gradient (loss of total proteins, especially albumin), and an increase in peritoneal capillary permeability. This latter mechanism, plus the difference between lymph production and excretion (which favors the accumulation of exudate), explains some of the most notable causes of non-hypertensive ascites (according to the serum albumin in ascites gradient (SAAG)), including peritoneal carcinomatosis and tuberculosis. We present the case of a young man, originally from a tuberculosis endemic area, in whom the study of ascitic fluid guided the workup and the definitive diagnosis, which was unexpected for his age. Finally, a practical approach to non-hypertensive ascites is provided.

## Introduction

Treatment of the patient with ascites is based on the serum ascites albumin gradient (SAAG) in a simultaneous serum and paracentesis sample [1], which classifies ascites into two groups: those associated with underlying portal hypertension, with liver cirrhosis being the classic prototype (SAAG >1.1); or those with direct peritoneal involvement or reduced oncotic pressure (SAAG <1.1), such as neoplasms, infections, or hypoalbuminemic states (nephrotic syndrome, acute pancreatitis). This last group is also known as causes of non-hypertensive ascites. The causes of non-hypertensive ascites represent about 15% of all cases, with cancer being particularly important in this group, presenting as peritoneal carcinomatosis in up to 66% of patients, with an ominous prognosis, translated into a median survival between five and 10 months in the absence of treatment; according to the classical records, 82% of cases correspond to metastatic adenocarcinomas, with colorectal origin described in only 5% of cases [2].

With the exception of ovarian neoplasms, ascitic fluid cytology could be erroneous, since it has a sensitivity of 60% and a specificity of 100%, being positive in less than 10% of primary liver neoplasms or liver metastases, which can also show a SAAG >1.1, given the intrahepatic venous stasis secondary to extrinsic compression of the sinusoids; therefore, when these causes are suspected, it is suggested to perform a biopsy whenever possible [3,4]. A complete cell count should always be performed, in addition to microbiologic studies (gram stain, cultures) depending on each particular case, as the presence of malignancies does not rule out concomitant secondary bacterial peritonitis due to viscus perforation or any other infectious involvement of the peritoneum [5]. Hence, we present the case of a young man, with no personal or family history of colorectal cancer, who presented with extensive peritoneal carcinomatosis (omental cake) in the framework of upper rectal cancer, even when infectious causes, which were ruled out systematically, could explain the initial clinical picture given his epidemiological profile.

## Case presentation

A 21-year-old African American male, who came from the Carmen de Atrato region (Chocó, Colombia), with no personal or family history, was admitted to the emergency department due to five months of abdominal pain. The pain was diffuse, intermittent, and started on epigastrium, and then it was associated with bloating, early satiety, and transient constipation periods of up to one week, with no associated rectal bleeding or hematochezia. No fever, chills, weight loss, functional class, paroxysmal nocturnal dyspnea, orthopnea, or bendopnea was present.

On physical examination, the patient was in stable condition, no jaundice was observed, and the abdomen was swollen and tense, with painful palpation in the upper quadrants; furthermore, an ascitic wave was present, and dullness was heard on the flanks. No collateral circulation or masses were observed. There was no jugular ingurgitation and no relevant findings on rectal examination or genital examination. The patient denied recent trips or any drug intake. An abdominal ultrasonography was performed two weeks before admission, which showed perihepatic, perisplenic spaces, and paracolic gutters, with an estimated volume of 250 mL, suggestive of peritoneal carcinomatosis.

**Figure 1:**
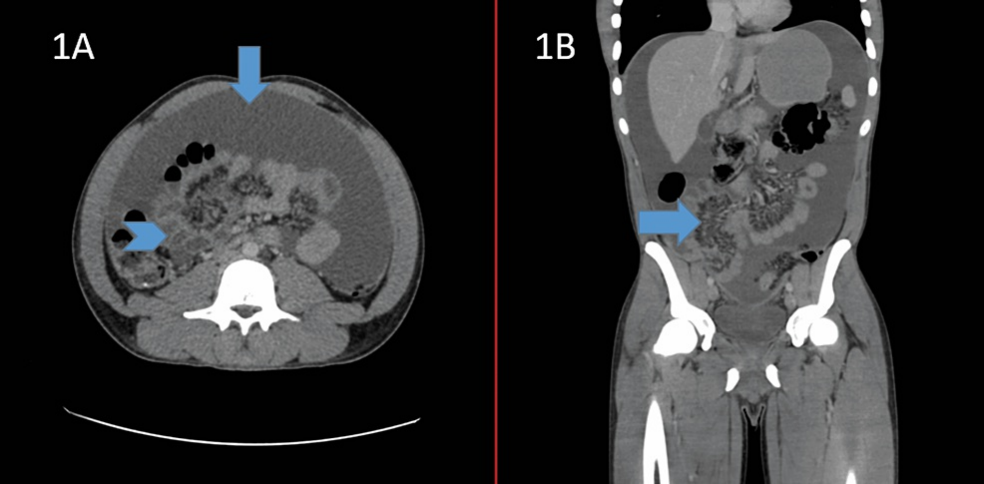
Simple and contrast abdomen and pelvis CT A. Axial view: abundant free liquid in the peritoneal cavity, with a central bowel pattern (arrow); nodular aspect of the omentum, with no focal lesions, and an increase in its vascularization due to multiple tubular structures (arrowhead). Thickened peritoneum, with up to 0.75 cm in the periphery. B. Coronal view: abundant free liquid in the abdominal and pelvic cavity, with bowel loops in mesogastrium, some of which are laid out in a radiated manner, with mesenterial hypervascularization in the center zone and the right iliac fossa (arrow).

On admission, hydration status was optimized, and analgesia was administered. Initial laboratory workup (Table 1) reported slight leukopenia and lymphopenia; no anemia or renal or hepatic dysfunction was observed. HIV enzyme-linked immunosorbent assay (ELISA) test was negative, as well as hepatotropic viruses. Given the ultrasound findings suggestive of peritoneal carcinomatosis, the patient was evaluated by General Surgery, which obtained a simple and contrasted computed tomography (CT) scan of the abdomen and pelvis (Figures 1A, 1B), in which, in addition to third-degree ascites, small bowel loops were observed stacked in the right iliac fossa, with an apparent granulomatous reaction; no hepatic or splenic lesions were observed, nor any abnormality in the pelvic image, with a normal splenoportal axis.

**Table 1:** Admission laboratory HBsAg: hepatitis B surface antigen, HCV Ab: hepatitis c virus antibody.

Laboratory	Result	Reference value
Complete blood count		
Hemoglobin	13.7 g/dL	13.5-18 g/dL
Hematocrit	42.8%	40%-54%
Leukocytes	4.42	4.5-11 x10^3 ^µL
Neutrophils	3.0	1.5-8 x10^3 ^µL
Lymphocytes	0.740	1.5-4 x10^3^ µL
Platelets	397	150-450 x10^3^ µL
Hepatic tests		
Alanine aminotransferase (ALT)	9.2 UI/L	10-49 UI/L
Aspartate aminotransferase (AST)	17.9 UI/L	0-34 UI/L
Alkaline phosphatase	70.1 UI/L	46-116 UI/L
Total bilirubin	0.67 mg/dL	0.30-1.20 mg/dL
Lactate dehydrogenase (LDH)	148.8 UI/L	120-246 UI/L
Albumin	4 g/dL	3.4-4.8 g/dL
Total protein	6.93 g/dL	5.70-8.20 g/dL
TP-INR	11.9-1.1	10.9
Infectious diseases		
HIV	0.050	Non-reactive (<1)
Thick blood smear	Negative	Negative
HBsAg	<0.10	Non-reactive (<1)
HCV Ab	0.13	Non-reactive (<0.8)
Tumoral markers		
Alpha-fetoprotein	<1.3	0-8
Carcinoembryonic antigen	Negative	Negative

**Figure 2:**
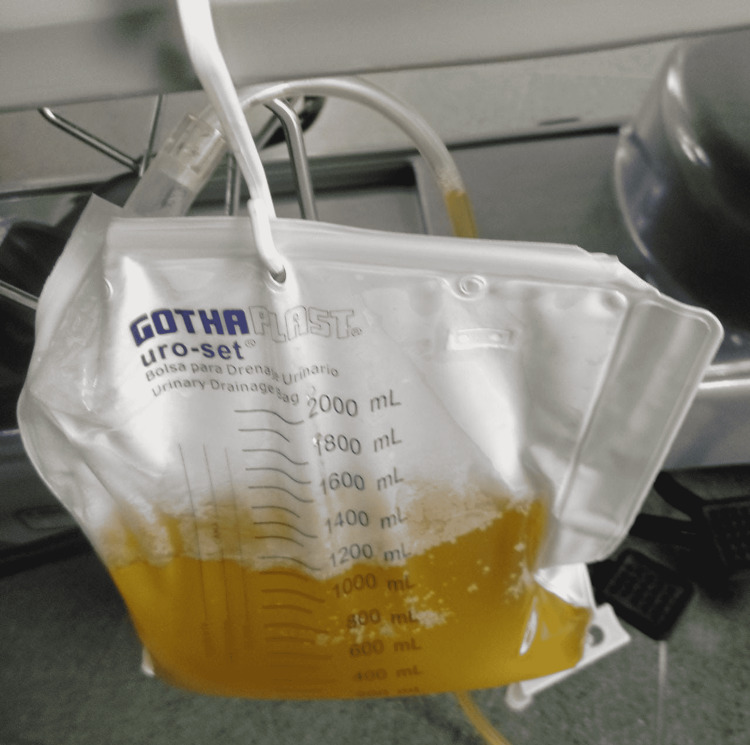
Ascitic fluid drained on diagnostic and therapeutic paracentesis

Moreover, the patient underwent a diagnostic and therapeutic paracentesis, obtaining 1,000 mL of clumpy liquid (Figure 2), with a dramatic improvement in abdominal pain. Ascitic fluid analysis was compatible with non-hypertensive ascites (SAAG: 0.99), in a young, immune-competent patient, with no evidence of solid organ parenchymal lesions, who came from an endemic zone for tuberculosis; hence, microbiological studies were performed, including culture and polymerase chain reaction for *Mycobacterium tuberculosis *(Table 2). A cellular block was processed, which was negative for malignancy.

**Table 2:** Ascitic fluid analysis LDH: lactate dehydrogenase.

Parameter	Result
Aspect	Clear yellow, with slight bloody sediment after centrifuge
Leukocytes	110/mm^3^: 80% neutrophils
Erythrocytes	50/mm^3^
Gram	Scarce leukocyte reaction, no bacteria. Wright Stain: 40% polymorphonuclear leukocytes, 60% mononuclear
Glucose	62.2 mg/dL (70-100 mg/dL)
Proteins	5.15 g/dL (0.3-4.1 g/dL)
Albumin	3.11 g/L (<11 g/L)
LDH	292.7 UI/L (<200 UI/L)
Amylase	50.7 mg/dL (138-404 mg/dL)
Adenosine deaminase (ADA)	13.99 UI/L (>45 UI/L)
Ascitic fluid smear	No acid-fast bacilli were observed.

**Figure 3:**
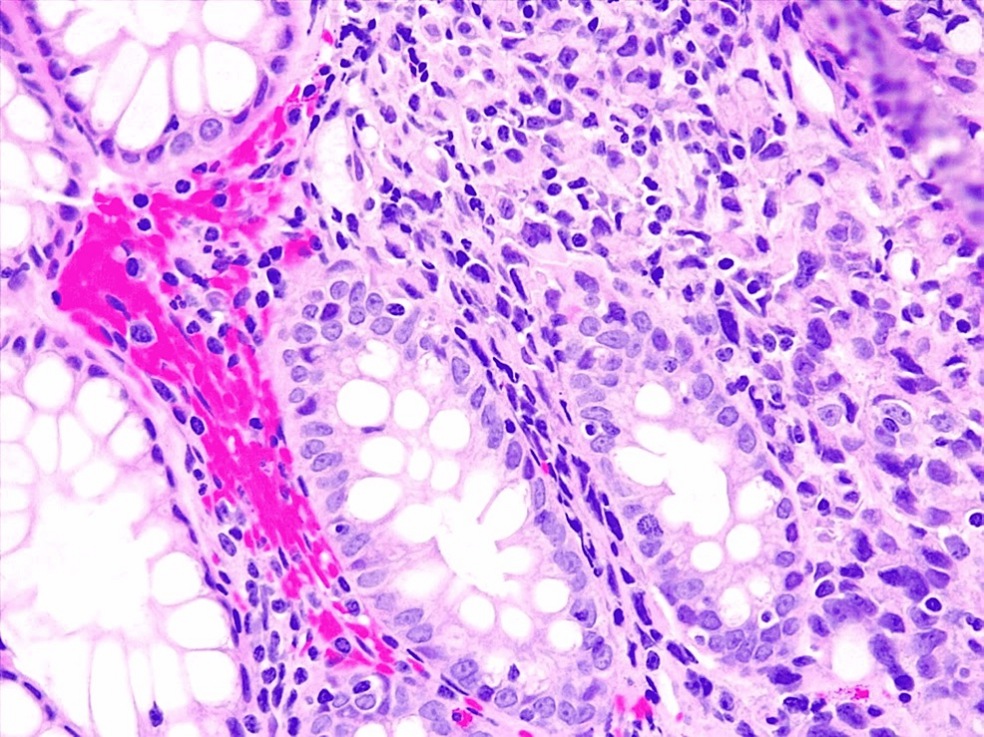
Biopsy of the superior rectum lesion, magnified 4x Superficial sample of colonic mucosae. Small foci of 0.8 cm wide located on the lamina propria, constituted by cells with enlarged nuclei, moderate pleomorphism, irregular borders, prominent nucleoli, and some atypical mitotic figures (wide and clear eosinophilic cytoplasm). Cells are arranged in a solid pattern, and no glandular structure is put together.

Therefore, a colonoscopy was performed to rule out gastrointestinal involvement by *M. tuberculosis.* However, the study reported an infiltrative, solid lesion on the superior rectum of 3 cm wide, with an erythematous surface and regular borders. A biopsy was taken, and the patient was discharged (Figure 3).

Two weeks later, the patient was readmitted for tension ascites and severe abdominal pain; on staging laparoscopy, 4,500 mL of ascitic fluid was drained, with evidence of infiltration of the omentum and mesogastrium, and a conglomerate of lesions of metastatic appearance in jejunum, ileum and colon, about 0.5 cm in diameter, with extensive involvement of the peritoneum and falciform ligament. A peritoneal carcinomatosis index (PCI) was calculated: 28 points from 13 abdomen-pelvis regions (Milan consensus, 2006). The preliminary report of peritoneum biopsy was compatible with moderately differentiated rectal adenocarcinoma, confirmed by immunohistochemistry (cytokeratin (CK) 7, CK20, and caudal homeobox protein 2 (CDX2)).

As a PCI higher than 20 is a contraindication for curative surgical intervention, a permanent drainage catheter was implanted in the abdominal cavity for recurrent ascites, and pain management was optimized by the palliative care team. Complementary studies, including simple and contrast thorax CT, ruled out distant metastases. Chemotherapy with palliative purpose was started by clinical oncology, with a CAPEOX/CAPOX protocol: capecitabine and oxaliplatin.

## Discussion

Colorectal cancer is the main gastrointestinal neoplasm, being the third in global incidence and the second cause of cancer death in the world. About 150,000 new cases are diagnosed annually in the United States, of which a minority (80 per year, or less than 1%) are adolescents and young adults. This figure has increased in the last 20 years, despite screening programs. The vast majority of these malignancies in this age group are sporadic [6].

The estimated lifetime risk is between 5% and 6%, being absolutely rare in kids and adolescents, with an annual incidence of 1/1,000,000 in the US population younger than 20 years; only 1% of all cases present before 30 years and 18% before 50 years. Of the latter group, 44% of cases with rectal cancer were observed in patients younger than 30 years [6,7]. In Colombia, colorectal cancer is the third leading cause of cancer death (9%, according to 2018 data), with 20%-25% of cases located in the rectum, with a higher risk of death in men, as in other latitudes. No age or region differences have been observed [8].

A debut with ascites usually involves an adverse prognosis. For example, the apparition of ascites on cirrhotic patients is associated to a mortality of 15% and 44% on one- and five-year follow-up, respectively [9], which is not different from an oncologic patient. It is estimated that 10%-15% of patients with colorectal cancer present with peritoneal carcinomatosis at the same time as primary tumor diagnosis. In addition, between 40% and 60% of cases with active treatment will present a relapse, with an exclusive peritoneal involvement in up to 20%-40% of patients [10].

Three main metastatic dissemination routes have been described for colorectal cancer: a lymphatic route, with dissemination from the tumor, with embolism which then deposits in lymph nodes; hematogenous route, which relates to venules involvement, generating distant deposits on liver and lung; and the third route is cell pass and peritoneal invasion, which may be due to tumor extension, with involvement of the whole wall, or peritoneal involvement after surgery due to trauma, blood loss, or lymphatic trauma. This explains that peritoneal carcinomatosis due to colon cancer may be the first step in the extension of the disease and not necessarily a consequence of established metastatic disease in the medium to long term [9,10].

In a retrospective study of 3,019 patients with colorectal cancer, 11% had peritoneal carcinomatosis. From this group, 61% had synchronous disease and 39% had metachronous disease. The most frequent site of appearance was the liver (15%). Generally, synchronous peritoneal metastatic presentation was 8%, and 25% for relapse [11]. Hereafter, some important elements to perform a differential diagnosis of non-hypertensive ascites are developed.

Ascitic fluid appearance

Peritoneal fluid is clear or pale yellow. Abdominal malignancies may develop chylous or hematic fluid (80% of patients with chylous ascites have cancer) [5,12]. Some benign tumors may also present with hematic fluid, such as Meigs syndrome (ovary fibroma or thecoma), perforated peptic ulcer, or acute hemorrhagic pancreatitis [13].

If the ascitic fluid is hemorrhagic (>10,000 red blood cells/μL), it must be corrected by subtracting one neutrophil for every 250 RBCs. For culture, inoculation must be performed on two blood culture flasks, with a sterile needle to avoid contamination. This procedure significantly improves its sensitivity. The rest of the fluid must be extracted on dry tubes or ethylenediaminetetraacetic acid (EDTA) tubes for cell count and biochemistry analysis, which is extrapolated from cirrhotic patients with spontaneous bacterial peritonitis [14]. Ascitic fluid analysis should be done as early as possible, preferably at the patient's bedside [15].

Ascitic fluid analysis

Total protein count from ascitic fluid does not differentiate completely between potential causes of ascites. On one side, Gupta et al. [16] observed that 24% of patients with uncomplicated cirrhosis had a total protein count higher than 2.5 g/dL; on the other hand, Alexandrakis et al. [17] observed that 20% of cases of malignant ascites had a low protein concentration, less than 2.5 g/dL. It has been observed that, according to the transudate/exudate distinction, only in 55% of cases, the etiology of ascites is correctly classified; nevertheless, SAAG correctly classifies the etiology of ascites in up to 97% of cases, even in the scenario of causes not associated to portal hypertension [18].

However, the interpretation of biochemical variables among cell count will allow performing specific diagnoses. Hence, a lymphocyte cell count, with proteins >2.5 g/dL and lactate dehydrogenase (LDH) >90 U/L, suggests peritoneal tuberculosis [19,20]. Adenosine deaminase (ADA) measurement on ascitic fluid >39 U/L predicts the diagnosis of peritoneal tuberculosis with a high sensitivity (100%) and specificity (97%) [20]. Nonetheless, diagnostic certainty will be obtained after microbiological and histological studies of peritoneal samples obtained through exploratory laparoscopy.

Specific scenarios on non-hypertensive ascites

The term malignant ascites is not a synonym for peritoneal carcinomatosis; the latter is the most common clinical presentation of the former (between 50% and 60% of cases). Other etiologies, in order of frequency, are grouped under the name of malignancy-related ascites: massive hepatic metastases, peritoneal carcinomatosis and massive hepatic metastases, hepatocarcinoma and hepatic cirrhosis, chylous ascites (usually secondary to lymphoma), and Budd-Chiari syndrome due to malignant occlusion of hepatic vein (Table 3) [19,20].

**Table 3:** Causes of non-hypertensive ascites Modified from Tarn and Lapworth [5]. *The presence of large mucus or mucin accumulation (sometimes with no cellularity, sometimes with cellularity and atypia, or even malignancy) in the peritoneal cavity, which may be associated to dissemination due to tumor rupture with a large mucinous component. These are mostly appendiceal mucinous tumors (cystadenoma or cystadenocarcinoma), and sometimes, ovarian cystadenomas with a large mucinous component.

Cause		Specific entities
Malignancies	Adenocarcinoma*	Ovary, breast, stomach, colorectal, endometrium, pancreas, lung
	Epidermoid carcinoma	Cervix; other epidermoid carcinomas
	Other carcinomas	Urothelial, hepatocellular, cholangiocarcinoma
	Non-epithelial	Melanoma, sarcoma, germ cell, mesothelioma
Infections	Mycobacteria	*Mycobacterium tuberculosis*, *M. avium* complex
	Fungi	*Histoplasmosis *spp., *Cryptococcus *spp., *Candida *spp.
	Parasite	*Toxocara *spp., *Strongyloides stercoralis*, *Giardia *spp., *Trichuris trichiura*, *Ascaris lumbricoides*, *Enterobius vermicularis*
	Bacteria	*Chlamydia trachomatis*, *Tropheryma whipplei*
Miscellaneous	Chylous ascites, pancreatic ascites, bilious ascites	
	Systemic lupus erythematosus (SLE), sarcoidosis, hypothyroidism	
	Ovarian disease	Meigs syndrome, struma ovarii, ovarian hyperstimulation syndrome, peritoneal pseudomyxoma
	Hypoalbuminemic states	Nephrotic syndrome, protein-caloric malnutrition, nephrogenic ascites (associated to hemodialysis)

It has been observed that around 18% of patients with hepatocarcinoma may present with a neutrophil cell count on ascitic fluid >250/μL. In these cases, a high red blood cell count >10,000/μL, as well as an RBC/WBC index >100 and a percentage of polymorphonuclear cells >75%, reasonably rules out concomitant active infection [18]. On the other hand, peritoneal carcinomatosis almost always presents with elevated cholesterol values (>50 mg/dL). When a malignant process is suspected, an ascitic fluid sample must be sent for cytological analysis, which has a sensitivity of 60% and a specificity close to 100%, according to some registries (performed mainly on ovarian cancer patients) [19,20].

The literature has described a few case reports such as ours. Of note is the report of a case in which a 27-year-old male from Uganda presented with severe abdominal distention, weight loss, and fever, clinical condition similar to that described in our case. Due to his place of provenance, tuberculosis, acute/chronic hepatitis, and lymphoproliferative disorders, as well as human immunodeficiency virus (HIV), were ruled out. Finally, a colorectal carcinoma was diagnosed from a cell block, showing atypical glands with an overabundance of extracellular mucin, suggestive of mucinous adenocarcinoma, which was confirmed by immunohistochemistry (strong positivity for CDX2, confirming a colorectal origin) [17]. Finally, management alternatives such as cytoreduction surgery and intraperitoneal hyperthermic chemotherapy have been tested on patients with peritoneal carcinomatosis of colorectal origin, with variable results in selected cases according to the prognostic factors widely validated [18,20].

## Conclusions

SAAG may be useful to differentiate whether ascites is secondary or not to portal hypertension (SAAG >1.1 g/dL vs < 1.1 g/dL, respectively), with a sensitivity of 95%. This way, in those patients with peritoneal carcinomatosis, with no liver lesions, SAAG will usually be <1.1 g/dL; in those with hepatic lesions (primary malignancies like hepatocarcinoma or metastatic lesions), SAAG will usually be >1.1 g/dL. The association of the clinical findings with the biochemical properties of the ascitic fluid, in addition to microbiological studies, will allow an adequate differential diagnosis, beyond the principle of parsimony, which holds that "all things being equal, the simplest of the competing explanations is the most likely to be correct"; it should not be forgotten that cancer is a rule-out diagnosis for non-hypertensive ascites, colorectal cancer being an increasing entity in the young population.
